# Heat shock response regulates stimulus-specificity and sensitivity of the pro-inflammatory NF-κB signalling

**DOI:** 10.1186/s12964-020-00583-0

**Published:** 2020-05-24

**Authors:** Anna Paszek, Małgorzata Kardyńska, James Bagnall, Jarosław Śmieja, David G. Spiller, Piotr Widłak, Marek Kimmel, Wieslawa Widlak, Pawel Paszek

**Affiliations:** 1 Department of Systems Biology and Engineering, Silesian University of Technology, Gliwice, Poland; 2 System Microscopy Centre, School of Biology, Faculty of Biology, Medicine and Health, University of Manchester, Manchester Academic Health Science Centre, Manchester, UK; 3 Maria Skłodowska-Curie National Research Institute of Oncology, Gliwice Branch, Gliwice, Poland; 4 Departments of Statistics and Bioengineering, Rice University, Houston, TX USA

**Keywords:** Heat-shock, HSF1, NF-kappaB signalling, IKK signalosome, Single-cell analyses, Live-cell imaging, Mathematical modelling

## Abstract

**Background:**

Ability to adapt to temperature changes trough the Heat Shock Response (HSR) pathways is one of the most fundamental and clinically relevant cellular response systems. Heat Shock (HS) affects the signalling and gene expression responses of the Nuclear Factor κB (NF-κB) transcription factor, a critical regulator of proliferation and inflammation, however, our quantitative understanding of how cells sense and adapt to temperature changes is limited.

**Methods:**

We used live-cell time-lapse microscopy and mathematical modelling to understand the signalling of the NF-κB system in the human MCF7 breast adenocarcinoma cells in response to pro-inflammatory Interleukin 1β (IL1β) and Tumour Necrosis Factor α (TNFα) cytokines, following exposure to a 37–43 °C range of physiological and clinical temperatures.

**Results:**

We show that exposure to 43 °C 1 h HS inhibits the immediate NF-κB signalling response to TNFα and IL1β stimulation although uptake of cytokines is not impaired. Within 4 h after HS treatment IL1β-induced NF-κB responses return to normal levels, but the recovery of the TNFα-induced responses is still affected. Using siRNA knock-down of Heat Shock Factor 1 (HSF1) we show that this stimulus-specificity is conferred via the Inhibitory κB kinase (IKK) signalosome where HSF1-dependent feedback regulates TNFα, but not IL1β-mediated IKK recovery post HS. Furthermore, we demonstrate that through the temperature-dependent denaturation and recovery of IKK, TNFα and IL1β-mediated signalling exhibit different temperature sensitivity and adaptation to repeated HS when exposed to a 37–43 °C temperature range. Specifically, IL1β-mediated NF-κB responses are more robust to temperature changes in comparison to those induced by TNFα treatment.

**Conclusions:**

We demonstrate that the kinetics of the NF-κB system following temperature stress is cytokine specific and exhibit differential adaptation to temperature changes. We propose that this differential temperature sensitivity is mediated via the IKK signalosome, which acts as a bona fide temperature sensor trough the HSR cross-talk. This novel quantitative understanding of NF-κB and HSR interactions is fundamentally important for the potential optimization of therapeutic hyperthermia protocols.

**Video Abstract**

## Plain English summary

Temperature changes affect how cells respond to their environment and have important clinical applications when sensitising cancer cells to treatment. However, our current understanding of how cells sense and adapt to elevated temperature is limited. In this work, we used live-cell imaging and mathematical modelling to understand the signalling of the NF-κB system, a critical regulator of inflammation and proliferation, following exposure to a range of physiological and clinical temperatures. We show that exposure to elevated temperature inhibits NF-κB signalling, but the level of the inhibition and the timing of their recovery is fine-tuned to specific signals and temperatures. In particular, we find that NF-κB responses to proinflammatory cytokine IL1β return to normal levels after 4 h, while TNFα-induced responses are more sensitive to temperature changes and their recovery is delayed. Using mathematical modelling and experimental perturbation we show that these responses are conferred via the crosstalk between HSF1 factor and IKK signalosome, a multiprotein kinase complex required for NF-κB activation. Our data predict that IKK acts as a bona fide temperature sensor through temperature-dependent denaturation and recovery, with the latter mediated via the heat-shock response pathways. Overall, we uncover stimulus specificity and sensitivity of the NF-κB system to elevated temperatures. Our results indicate that TNFα signalling is inhibited by elevated temperatures more effectively than IL1β signalling. This would be important for more efficient therapeutic hyperthermia protocols but may also have physiological consequences during fever.

### Background

The evolutionary conserved Heat Shock Response (HSR) system regulates how cells adapt to stress [1]. HSR involves a large family of molecular chaperons called Heat Shock Proteins (HSP) produced as internal repair mechanisms against thermal damage [2, 3]. In eukaryotic cells, the HSR response is regulated transcriptionally by the Heat Shock Factor 1 (HSF1) [4]. In resting cells, HSF1 monomers are kept in an inactive form via their association with heat shock proteins [5]. High temperatures cause protein conformational changes that in turn leads to a redistribution of HSPs from complexes with HSF1 towards the damaged proteome to initiate repair [6]. This results in the release of HSF1 monomers and their activation via trimerization and posttranslational modifications [7, 8]. The activated HSF1 transcription factor then leads to the production of different HSP family members. HSPA1 (HSP70) is thought to be the most robustly activated by HSF1, while others, including HSP90, the main inhibitor of HSF1 activity, are present at a relatively constant level [5]. HSPs restore protein homeostasis via de novo protein folding and targeted degradation and eventually inhibit HSF1 activity [9]. They also induce a state of thermotolerance limiting further damage to repeated HS [10].

Evolutionary conserved NF-κB regulates the expression of hundreds of genes involved in inflammation as well as control of apoptosis, proliferation, cell adhesion and aging [11]. The NF-κB family includes five proteins, all characterised by the presence of the Rel Homology Domain responsible for dimerization as well as DNA and protein binding, but the ubiquitously expressed p65/p50 heterodimer is thought to be most abundant [12]. The NF-κB system integrates a variety of signals, including pro-inflammatory cytokines, such as Tumour Necrosis Factor α (TNFα) and Interleukin 1β (IL1β), bacterial products, viruses, foreign DNA/RNA and many others [13]. Selective signal integration relies on activation of the inhibitory κB kinase (IKK), a multiprotein signalosome complex composed of catalytic IKKα and IKKβ as well as regulatory IKKγ subunits, required for phosphorylation-mediated degradation of the Inhibitory κB proteins (IκB) and subsequent NF-κB translocation into the nucleus [14–17]. In resting cells, NF-κB is sequestered in the cytoplasm by association with IκB, but upon stimulation undergoes nuclear-to-cytoplasmic oscillations as a result of cyclic degradation and NF-κB-dependent resynthesis of IκB and A20 (another regulator of NF-κB) [18, 19]. The patterns of NF-κB nuclear translocations were shown to control target gene expression [20–23], which may confer both proapoptotic or prosurvival functions [12]. NF-κB signalling plays a key role in disease, particularly in cancer progression [24]. In breast cancer, the NF-κB activity stimulates tumour growth, metastasis, and chemoresistance, therefore therapeutic inhibition of its activity is considered beneficial [25].

Hyperthermia, the exposure of tissue to high temperature, has been considered a promising strategy to sensitise cancer cells to therapeutic intervention [26]. However, a better understanding of the temperature effect on cellular signalling, in general, and crosstalk between HSR and NF-κB systems, in particular, is required for the optimization of such treatment. The NF-κB system can adapt to physiological (< 40 °C) temperatures [27], but exposure to higher temperatures (> 40 °C) results in the attenuation of the NF-κB signalling and function [25, 28–30]. This involves IKK denaturation [28, 31–33], with inhibition of IκB degradation, NF-κB phosphorylation and translocation as well as target gene expression [29, 30, 34–38]. The mechanistic understanding of the underlying processes is confused with conflicting findings and lack of quantitative analyses. For example, HSPA1 and HSP90 were shown to repair and stabilize IKK [39, 40], while overexpression of HSPA1 was found to inhibit IKK activity [41] and TRAF2-mediated NF-κB activation [42]. Here we use interdisciplinary systems biology approaches to systematically understand how elevated temperature affects NF-κB signalling to cytokine treatment using live single-cell imaging and mathematical modelling.

## Methods

### Cell culture and reagents

Experiments were performed using the human MCF7 adenocarcinoma cell line (purchased from ATCC®; cat. no. HTB-22™). Cells were cultured at 37 °C in humidified 5% CO_2_ in DMEM/F12 medium (Gibco) supplemented with 10% (v/v) heat-inactivated fetal calf serum and routinely tested for mycoplasma contamination. For confocal and Western blotting experiments, HS response was induced by transferring cells into a water bath at 43 °C (unless otherwise stated). After 1 h of HS, cells were supplemented with fresh 37 °C media. 10 ng/ml of human recombinant TNFα or IL1β (Calbiochem) was used to simulate NF-κB responses for the indicated time periods.

### Engineering of p65-EGFP and HSF1-dsRed stably transfected MCF7 cell line

The p65-EGFP sequence was re-cloned from p65-EGFP-N1 plasmid [18] into pLNCX2 vector (Clontech) for expression under a CMV promoter (using *HindIII* and *Not1* restriction sites). HSF1 coding sequence was amplified by PCR on cDNA template, cloned in frame in dsRed-N1 plasmid and then HSF1-dsRed was re-cloned into pLNCX2 vector (using *AgeI* and *NotI* restriction sites). The Retroviral Gene Transfer protocol (Clontech) was followed to obtain a stable MCF7 line expressing the resulting p65-EGFP-pLNCX2 or HSF1-dsRed-pLNCX2 vector. In brief, RetroPack™ PT67 packing cells were transfected with p65-EGFP-pLNCX2 or HSF1-dsRed-pLNCX2 vector using TurboFect™ (Thermo Scientific), then cells were selected with G418 geneticin sulphate (Gibco), and the virus-containing medium was collected after 1 week of culture. MCF7 cells were exposed to the virus-containing medium and transduced cells were sorted based on EGFP or dsRed expression.

### Protein extraction and Western blotting

Cells were lysed in 1% NP-40, 0.5% sodium deoxycholate and 0.1% SDS in PBS, supplemented with Complete™ (Roche) protease and phosphatase inhibitor cocktail, centrifuged for 20 min at 14,000 rpm at 4 °C. The supernatants, defined as a soluble fraction, were collected. Insoluble proteins, remaining in the pellets, were dissolved in an SDS sample buffer consisting of 25 mM Tris-HCl pH 6.8, 0.5% SDS, 2.5% glycerol, and 15% 2-mercaptoethanol and sonicated (30 times 30s). Protein concentration was determined by BCA assay (Thermo Scientific). Samples and ladder (Bio-Rad, #161–0375) were resolved on polyacrylamide gels and transferred to nitrocellulose membranes (Amersham), incubated 1 h at room temperature in blocking buffer (5% (w/v) non-fat milk powder in TBS-T containing 0.25 M Tris–HCl pH 7.5, 0.1% Tween-20, 0.15 M NaCl), washed 3 times in TBS-T and incubated overnight with primary antibody (p65-S536, CST #3033; IKKα, CST #11930S; IKKβ, CST #2684S; HSF1, CST #4356; HSF1-S326 Abcam ab76076; HSPA1, Stressgen ADI-SPA-810-D; β-actin, Sigma-Aldrich A3854) at 1:1000 dilution in blocking buffer. Membranes were washed 3 times in TBS-T and incubated with 1:1000 HRP-conjugated secondary antibody for 1 h at RT. Membranes were washed (3 x TBS-T) then incubated with Luminata Crescendo Western HRP Substrate (EMD Millipore Corp.) and the signal was detected by exposure to Carestream Kodak BioMax MR film (Sigma-Aldrich). Densitometric analyses of blots were performed using Image Studio Lite software to calculate relative protein expression after normalization with loading controls.

### HSF1 siRNA knock-down

MCF7 or p65-EGFP expressing cells were plated into 35 mm culture dishes 1 day before transfection. The transfection mix was prepared using DharmaFECT 1 Transfection Reagent (GE Dharmacon) according to the manufacturer’s protocol. Each dish was transfected with 100 nM of human HSF1 On-Target Plus siRNA or On-Target Plus non-targeting pool siRNA (both GE Dharmacon). Cells were cultured with the transfection mix for 48 h. After 48 h the entire procedure was repeated. The cell medium was replaced with a fresh one and a fresh transfection mix, then cells were used in further experiments after another 48 h.

### Gene expression analysis

Total RNA was extracted from wild type MCF7 cells using the Roche High Pure RNA Isolation Kit. SuperScript™ VILO™ kit (Invitrogen) was used for the production of cDNA. For each sample, 2 μg of RNA was used. The manufacturer’s protocol was followed. Briefly, the cDNA was diluted 1:20 with RNase free water. Quantitative RT-PCR was performed using Roche LightCycler® 480 Instrument II. A total of 5 pM of forward and reverse primers, cDNA template was added to the 2x LightCycler® 480 SYBR Green I Master (Roche). Primers used in the analyses are listed in Table S1. Relative quantification was used to calculate the fold difference based on the threshold cycle (CT) value for each PCR reaction using 2^-ΔΔCT^ method. The target gene was normalised to the reference gene *GAPDH*, with corresponding control used as the calibrator.

### Confocal microscopy

Cells were plated onto 35 mm-glass-bottomed dishes (Greiner Bio-One) 1 day prior to the experiment and incubated on the microscope stage at 37 °C in humidified 5% CO_2_. The Hoechst 33342 (Molecular Probes) staining was performed immediately before the experiment. Two Carl Zeiss confocal microscopes were used (LSM780, AxioObserver and LSM880 AxioObserver) with Plan-Apochromat 40x/1.4 Oil DIC M27 and Fluar 40x/1.30 M27 Oil objectives. The 488 nm (ATOF set at 4%) line from an argon ion laser was used to excite the p65-EGFP fusion protein and emitted light between 498 and 598 nm was detected through pinholes set to 5 μm. The 405 nm (ATOF set at 1%) line from a diode laser was used to excite Hoechst 33342 and emitted light 410–490 nm was detected. The 556 nm (ATOF set at 2%) line from a diode laser was used to excite the HSF1-dsRed fusion protein and emitted light 580–650 nm was detected. For the series of interrelated confocal experiments, the same microscope settings have been used. Image capture was performed using the Zeiss Zen 2010b or Zen2 software. Quantification of p65-EGFP nuclear fluorescence was performed using automated segmentation and tracking of Hoechst-labelled cell nuclei with Cell Tracker (version 0.6) [43] and in-house software. The data was exported as mean fluorescence intensity. Trajectories of the nuclear p65-EGFP were normalized across presented conditions and displayed as heat maps. HSF1-dsRed granule quantification was performed in CellProfiler with a modified speckle counting pipeline [44].

### Evaluation of TNFα and IL1β internalization

MCF7 cells were plated onto 4-compartment 35 mm-glass-bottomed imaging dishes (Greiner Bio-One) in culture medium 1 day prior to the experiment and incubated at 37 °C in humidified 5% CO_2_ on the microscope stage. Cells were treated with human recombinant TNFα or IL1β biotin conjugate (1 μg/ml, Fluorokine, R&D Systems, Wiesbaden) diluted to 10 ng/ml in 20 μl of avidin-FITC (10 μg/ml) and made up to 50 μl with a minimum essential medium. Carl Zeiss LSM880, AxioObserver confocal microscope with a Plan-Apochromat 40x/1.4 Oil DIC M27 objective was used with 488 nm excitation and 493-634 nm emission signal detection. Image capture was performed using Zeiss Zen 2 software to take time-lapse 13 deep Z stacks over 13 μm with a 1 Airy unit pinhole diameter. Maximum intensity projections were used for image analysis.

### Statistical analyses

Statistical analyses were performed in GraphPad Prism 7.02. Normal distribution was assessed with D’Agostino-Pearson test. Nonparametric tests were applied for non-normal distribution data. Kruskal-Wallis one-way ANOVA with Dunn’s multiple comparisons was used for characteristics of single cell NF-κB responses. Differences in the percentage of responding cells were assessed with Chi-square test.

### Mathematical modelling

We considered simplified models of HS-induced HSPi protein accumulation [7, 45–48] combined with a previously published model of TNFα and IL1β-dependent NF-κB signalling [15]. Models were fitted to recapitulate: (1) HSF1 activation (via release from the HSP-HSF1 complex due to protein denaturation) and accumulation of HSPi proteins (via transcriptional regulation with a Hill coefficient *n* = 3 corresponding to HSF1 trimerization). (2) MCF7-specific NF-κB dynamics; namely, the dampening and low first peak amplitude of NF-κB oscillations in response to TNFα was recapitulated by a reduced IκBα transcript rate [49] and IKKK_TNF_ activation rate, while a single translocation in response to IL1β by a slower rate of IKKK_IL1_ cycling (in comparison to IKKK_TNF_). Subsequently, the HSR and NF-κB cross-talk was introduced in the combined model by assuming (1) temperature nonlinearly affects denaturation rate of IKK and IKKK kinases, and no other molecules in the system; (2) HSPs prevent denaturation of IKK and IKKK kinases [10]; (3) Repair of the TNFα receptor-associated kinase (IKKK_TNF_) requires HSPi; (4) Repair of IKK and IL1β receptor-associated kinases (IKKK_IL1_) involves HSPc; (5) HSPs accelerate the transition between active and inactive kinase states. Crosstalk parameters were fitted to recapitulate single cell NF-κB responses (to TNFα and IL1β stimulation) in wild-type and HSF1 knock-down cells at different times after HS as well as data on IKK denaturation and HSF1 activation. The final mathematical model includes 31 ordinary differential equations and 60 parameters that describe protein association/dissociation/degradation and mRNA transcription/translation using mass action kinetics (see Fig. 4c, d and S5A, B for model fits, Table S2, S3 and S4 for model variables, differential equations and parameter values). Model simulations were divided into three phases: (i) heat shock - 1 h HS at 43 °C (Fig. 4) and subsequently extended to 38–43 °C range (Fig. 5, S6 and S7), (ii) recovery time - up to 8 h at 37 °C, (iii) TNFα or IL1β stimulation for 10 h at 37 °C. Heat shock treatments were subsequently repeated to test the effect of thermotolerance (Fig. S9). In order to simulate heterogeneous cell responses, random initial numbers of molecules for IKK and IKKK, as well as HSPc and HSF1 were assumed. Initial conditions were drawn from the log-normal distribution with parameters μ = 11.5 and σ = 0.15 for IKK, IKKK, μ = 10.1 and σ = 0.15 for HSPc, and μ = 9.2 and σ = 0.15 for HSF1 molecules. In simulations with HSF1 knock-down, a 95% reduction in the amount of HSF1 molecules was assumed. Sensitivity analyses were performed by varying temperature (by 1 °C) and parameter values (one parameter at a time over an 8-fold range comparing with the nominal value for a given cytokine transduction pathway), results were presented as heat maps (Fig. S8). Simulations were performed in MATLAB R2016a with ‘ode15s’ function.

## Results

### HS modulates the NF-κB response to TNFα stimulation

**Fig. 1:**
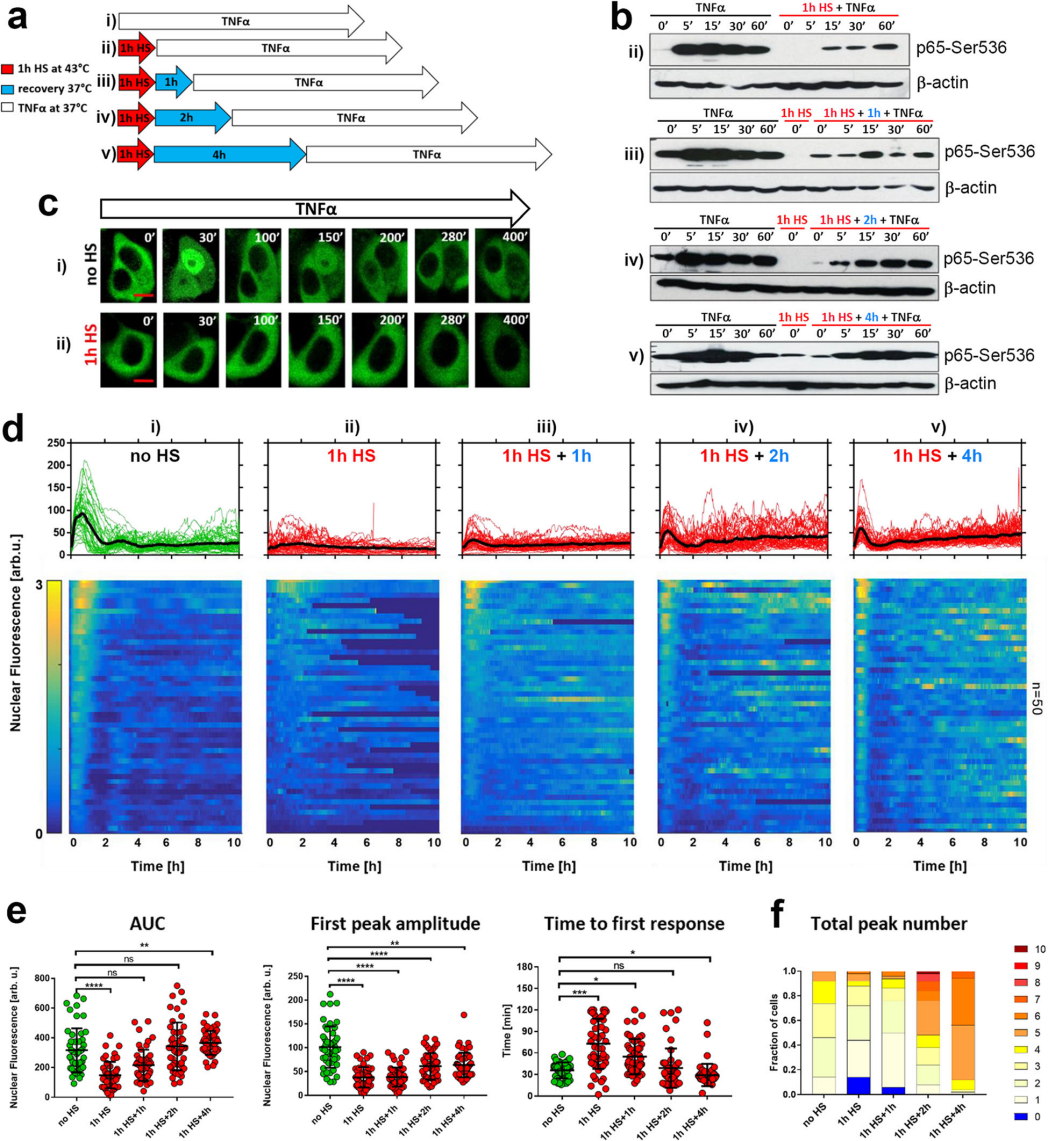
Heat shock modulates the NF-κB responses to TNFα stimulation. **a** Schematic representation of HS and TNFα treatment: MCF7 cells were either cultured under normal conditions (37 °C) (i) or subjected to 1 h HS at 43 °C, recovered for 0, 1, 2, or 4 h (ii - v, respectively), and treated with TNFα. **b** The level of p65-Ser536 phosphorylation in response to HS and/or TNFα treatment (as represented in **a**) assayed via Western blotting in whole cell lysates at indicated time points (min). Shown also are cytokine-untreated controls (0′) and β-actin loading control. **c** Confocal microscopy images of representative cells stably expressing p65-EGFP. Top panel: cells cultured under normal conditions (at 37 °C, no HS) and stimulated with TNFα (displayed in minutes at indicated times). Bottom panel: cells exposed to 1 h HS at 43 °C and stimulated with TNFα (displayed post HS at indicated times). Scale bar 5 μm. **d** Nuclear NF-κB trajectories in cells stably expressing p65-EGFP for different HS and TNFα treatment conditions (as represented in **a**). Top: individual single cell trajectories (*n* = 50 per condition, in aribirary fluorescene units) depicted with green or red lines; black lines represent the population averages. Bottom: heat maps of single cell trajectories normalized across all considered conditions (represented by an arbitrary 0–3 scale). Cells monitored for up to 10 h from the beginning of TNFα stimulation. **e** Characteristics of single cell nuclear NF-κB trajectories presented in **d**. From the left: distribution of area under the curve (AUC), first peak amplitude, and time to first response. Individual cell data are depicted with circles (with mean ± SD per condition). Kruskal-Wallis one-way ANOVA with Dunn’s multiple comparisons test was used to assess differences between groups (*p < 0.05, **p < 0.01, ***p < 0.001, ****p < 0.0001*,* ns – not significant). **f** Distribution of the total peak numbers between different experimental conditions

Previously we showed that elevated temperature inhibits NF-κB signalling in responses to TNFα stimulation [28]. Here we sought to investigate the long-term modulation and recovery of NF-κB responses following HS. We used a treatment protocol where breast adenocarcinoma MCF7 cells were exposed to 1 h 43 °C HS, subsequently recovered in normal conditions (37 °C) for up to 4 h and then treated with 10 ng/ml TNFα (Fig. 1a). This defines a “recovery time” between the end of HS exposure and the cytokine treatment, which is relevant to therapeutic hyperthermia protocols [46, 50]. In cells maintained under normal conditions, we observed a robust Ser536 p65 phosphorylation, a marker of NF-κB activity, which peaked around 15 min after TNFα stimulation (Fig. 1b). In contrast, stimulation immediately after 1 h HS exposure did not result in rapid Ser536 p65 phosphorylation. The response was inhibited and delayed in time. Following extended recovery times, Ser536 p65 phosphorylation steadily increased. HS exposure may lead to heterogenous NF-κB responses in single cells, which can be masked in the population level analyses [28]. Therefore, we developed a MCF7 cell line stably expressing p65-EGFP suitable for quantitative single-cell time-lapse microscopy analyses. We confirmed, that in the engineered cells the TNFα-mediated behaviour of the Ser536 p65-EGFP was consistent with that of the endogenous p65 (Fig. S1A). In addition, we confirmed that HS exposure alone did not cause p65-EGFP translocation (Fig. S1B). Using live single-cell microscopy, we assayed TNFα-induced responses after different HS recovery times in the fluorescent reporter line (see Fig. 1c-f for confocal images of representative cells and for analysis of single cell traces post HS). In agreement with previous imaging studies [51], including analyses of endogenous p65 [52], stimulation of MCF7 cells cultured in normal conditions resulted in a rapid nuclear translocation of p65-EGFP followed by series of damped oscillations. Individual cells exhibited varied amplitude of the first nuclear translocation (mean 101 arbitrary fluorescence units ±44 standard deviations) as well as a degree of damping. Approximately 50% of cells exhibited up to three nuclear NF-κB translocations, while no cells showed more than five translocations within the 10 h imaging window (see Fig. 1f for the distribution of the number of translocations). TNFα stimulation immediately after exposure to 1 h HS resulted in statistically significant inhibition of the p65-EGFP responses where cells exhibited only residual and delayed activity (Fig. 1d, as well as the reduced area under the curve, AUC, Fig. 1e). As the recovery time increased, we observed increases in the first peak nuclear p65-EGFP amplitude, which coincided with a shorter time to first response (Fig. 1e). However, even after a 4 h recovery period the initial NF-κB response remained suppressed compared to cells cultured in the normal conditions. Interestingly, the HS exposure induced the long-term oscillatory phase of the NF-κB response (as evident by heat-maps and the increased number of cells exhibiting multiple nuclear NF-κB translocations, Fig. 1f). After 4 h recovery, almost 90% of cells exhibited at least five nuclear translocations (and 50% after 2 h recovery) within 10 h after cytokine treatment. This is indicative of HS-mediated response modulation via intracellular signalling [27] rather than changes of external TNFα concentration over time [53].

In order to understand the functional consequence of the HS-mediated NF-κB inhibition, we measured the expression of NF-κB target genes by quantitative RT-PCR at 90 mins after TNFα stimulation (corresponding to the early activation phase, Fig. S1C). The analysis of the NF-κB negative inhibitor *TNFAIP3* and *NFKBIA* genes (coding for A20 and IκBα, respectively), as well as *TNF* and chemokine *CCL2* demonstrates a substantial suppression of the TNFα-induced mRNA expression after HS exposure. However, following the 4 h recovery time, the degree of their activation returned nearly to the levels observed in stimulated cells not subjected to heat shock.

### Recovery of the HS-modulated NF-κB response to cytokines is stimulus-specific

**Fig. 2:**
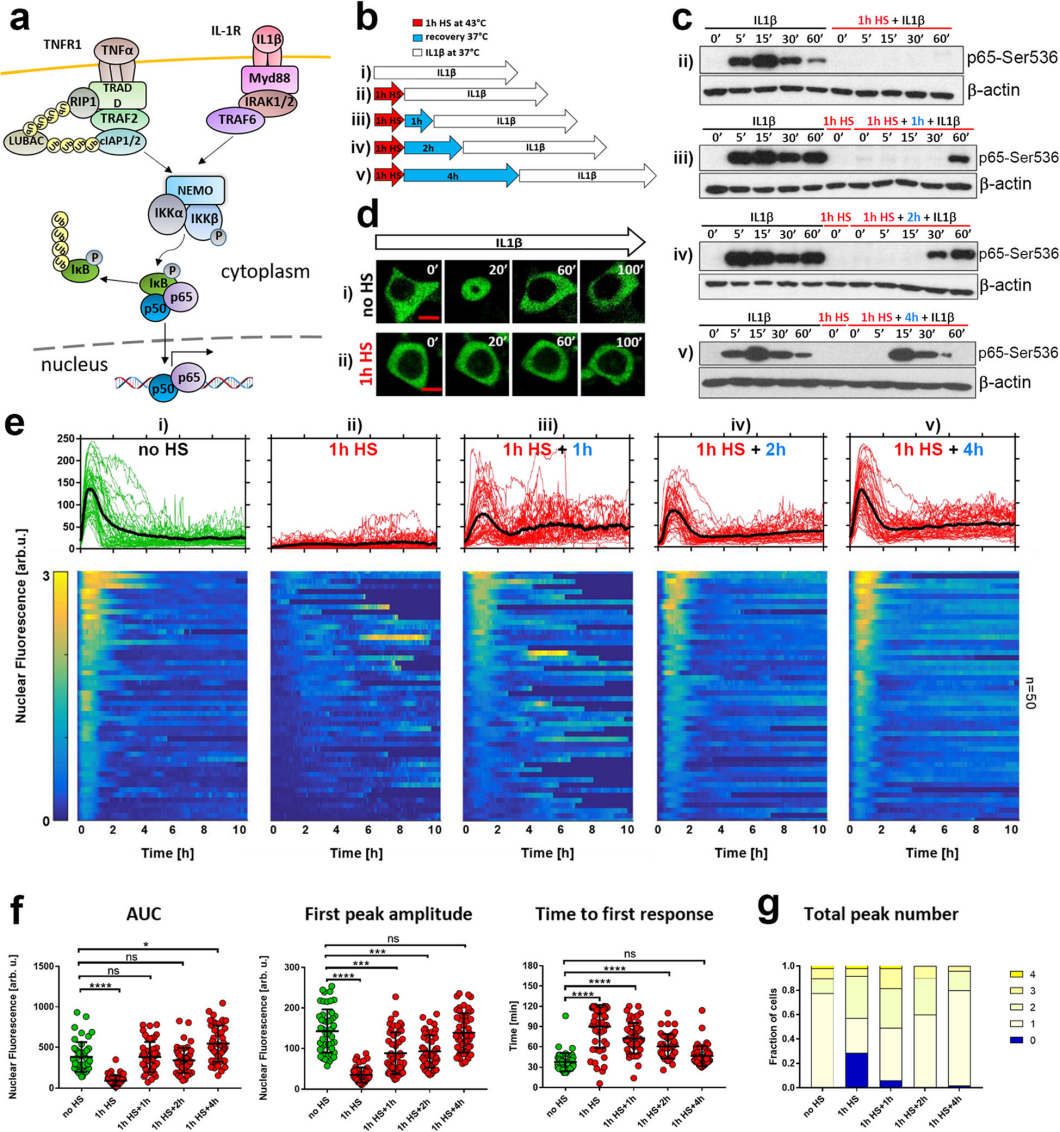
Heat shock modulates the NF-κB responses to IL1β stimulation. **a** Schematic diagram of TNFα and IL1β-dependent signal transduction pathways leading to IKK and NF-κB activation. **b** Schematic representation of HS and IL1β treatment: MCF7 cells were either cultured under normal conditions (37 °C) (i) or subjected to 1 h HS at 43 °C, recovered for 0, 1, 2, or 4 h (ii - v, respectively), and treated with IL1β. **c** The level of p65-Ser536 phosphorylation in response to HS and/or and IL1β treatment (as represented in **b**) assayed via Western blotting in whole cell lysates at indicated time points (min). Shown also are cytokine-unstimulated controls (0′) and β-actin loading control. **d** Confocal microscopy images of representative cells stably expressing p65-EGFP. Top panel: cells cultured under normal conditions (at 37 °C, no HS) and stimulated with IL1β (displayed at indicated times). Bottom panel: cells exposed to 1 h HS at 43 °C and stimulated with IL1β (displayed at indicated times post HS). Time after IL1β stimulation displayed in minutes. Scale bar 5 μm. **e** Nuclear NF-κB trajectories in cells stably expressing p65-EGFP for different HS and IL1β treatment protocols (as represented in **b**). Top: individual single cell trajectories (n = 50 per condition, in aribirary fluorescene units) depicted with green or red lines; black lines represent the population averages. Bottom: heat maps of single cell trajectories normalized across all considered conditions (represented by an arbitrary 0–3 scale). Cells monitored for up to 10 h from the beginning of IL1β stimulation. **f** Characteristics of single cell NF-κB trajectories from **e**. From the left: distribution of area under the curve (AUC), first peak amplitude, and time to first response. Individual cell data are depicted with circles (with mean ± SD per condition). Kruskal-Wallis one-way ANOVA with Dunn’s multiple comparisons test was used to assess differences between groups (*p < 0.05, ***p < 0.001, ****p < 0.0001*,* ns – not significant). **g** Distribution of the total peak numbers between different experimental conditions

TNFα acts through its cognate receptor to activate IKK, and a number of mechanisms have been proposed to understand how HSR affects this process [28, 41]. In the first attempt to investigate these mechanisms, we utilised IL1β cytokine, which is known to activate IKK via signal transduction pathway parallel to that of TNFα (Fig. 2a) [15–17, 54]. Using a treatment protocol analogous to that for TNFα (Fig. 2b) we assayed the early NF-κB response to 10 ng/ml IL1β treatment following HS at the population level (Fig. 2c). In cells maintained under normal conditions, IL1β treatment induced a rapid induction of Ser536 p65 phosphorylation, which was blocked immediately after HS exposure. However, IL1β-induced Ser536 phosphorylation levels appeared to return to the pre-HS level after 4 h recovery. We performed live single-cell microscopy experiments using the MCF7 cell line stably expressing p65-EGFP fusion protein after treatment with IL1β (Fig. 2d-g). Stimulation of cells maintained in normal conditions resulted in rapid and robust p65-EGFP translocation to the nucleus. In contrast to TNFα treatment, most of the cells (80%) exhibited only a single nuclear translocation, while the remaining 20% of cells exhibited up to four translocations (Fig. 2g). Transient responses to IL1β stimulation were also reported in fibroblast cells [16, 54], whereas other cell types may exhibit behaviour similar to that induced by TNFα [15]. IL1β treatment immediately after exposure to 1 h HS resulted in inhibition of the p65-EGFP response, although some cells exhibited residual but delayed activation (as revealed by a heat map, Fig. 2e). Responses to stimulation after different recovery times revealed a complete transition to pre-HS levels, which was exhibited by increasing first peak amplitude, AUC as well as reducing response time (Fig. 2f). In contrast to TNFα, HS exposure did not alter the long-term NF-κB dynamics, as the distribution of peak numbers was similar after 4 h recovery and in cells maintained under normal conditions (Fig. 1g). Functionally, HS inhibited NF-κB-dependent IL1β-stimulated transcription, which subsequently returned to the pre-HS levels after 4 h recovery (Fig. S1C).

Overall, these data demonstrate that while the HS exposure may effectively inhibit cytokine-mediated NF-κB signalling and gene expression responses, the kinetic of adaptation to HS during the recovery at physiological temperature is stimulus-specific.

### HSF1 differentially regulates stimulus-specific NF-κB response to cytokines

**Fig. 3:**
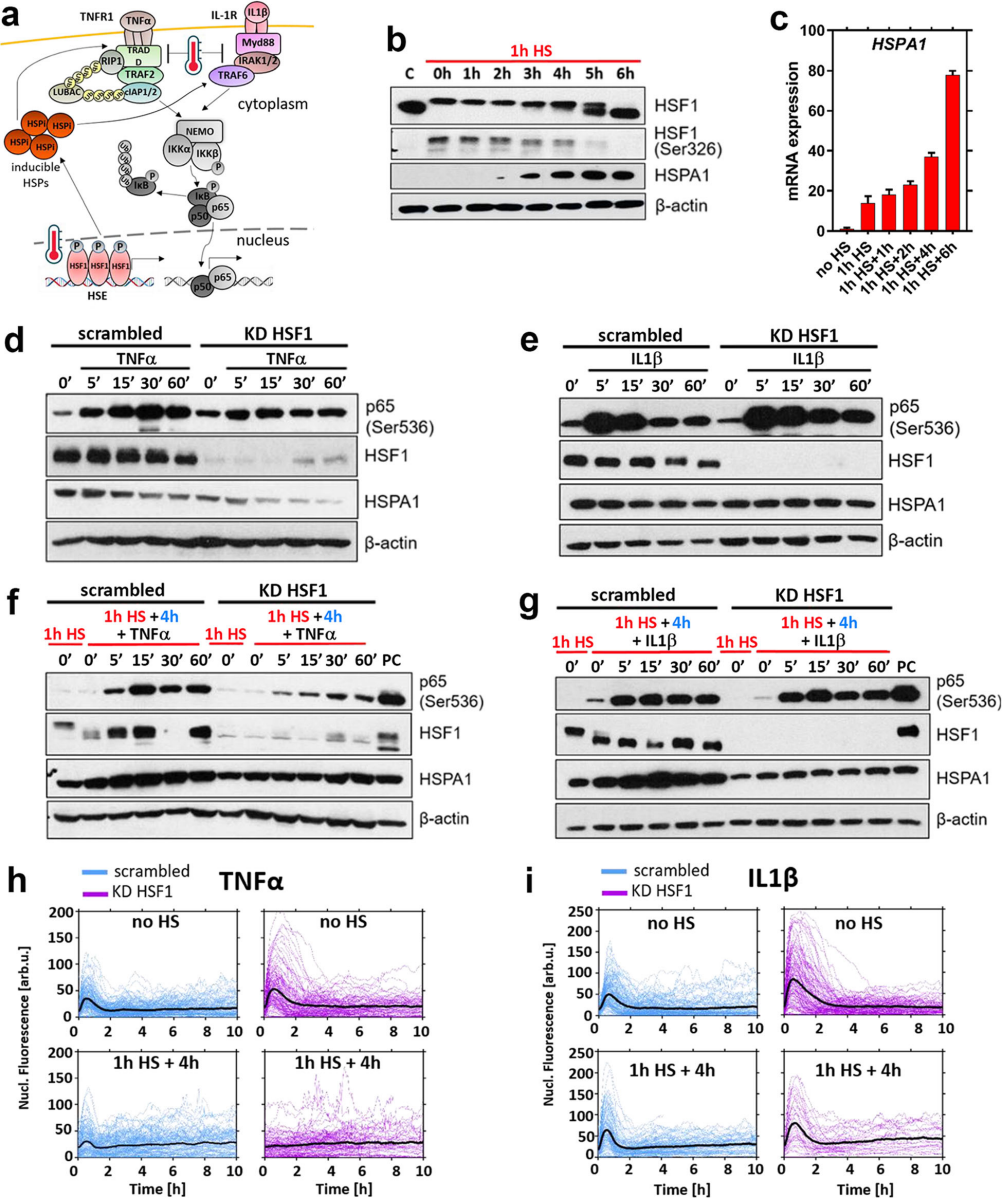
HSF1 regulates the recovery of TNFα-induced NF-κB signalling after HS. **a** Schematic representation of the HSR and NF-κB crosstalk. **b** Western blot analysis of HSF1, phosphorylated HSF1-Ser326 and HSPA1 proteins level in MCF7 cells. Cells were either cultured in normal conditions (C) or subjected to 1 h HS at 43 °C then recovered for up to 6 h. β-actin was used as a loading control. **c** Quantitative RT-PCR analysis of *HSPA1A* mRNA abundance at different time points after 1 h HS at 43 °C (normalised to the reference *GAPDH* gene)*.* Shown are fold-changes (mean ± SDs of three replicate experiments) with respect to expression at 37 °C (no HS)*.***d** Effect of HSF1 knock-down on TNFα-induced response. MCF7 cells cultured in normal conditions were treated with scrambled siRNA control (scrambled) and HSF1-specific siRNA (KD HSF1) and stimulated with TNFα for indicated time periods (min). Shown are the levels of p65-Ser536, HSF1 and HSPA1 assayed via Western blotting of the whole cell lysates. Shown are cytokine-unstimulated controls (0′) and β-actin loading control. **e** Effect of HSF1 knock-down on IL1β-induced response in cells cultured and stimulated as in **d**. **f** Effect of HSF1 knock-down on TNFα-induced response following 4 h HS recovery. Cells treated with scrambled siRNA control (scrambled) and HSF1-specific siRNA (KD HSF1) were subjected to 1 h HS at 43 °C and recovered for 4 h, then stimulated with TNFα for indicated times (in min). Shown are the levels of p65-Ser536, HSF1, and HSPA1 assayed via Western blotting of the whole cell lysates. Shown also are cytokine-unstimulated controls (0′) and β-actin loading control. As a positive control (PC), scrambled 15′ TNFα sample from **d** was loaded. **g** Effect of HSF1 knock-down on IL1β-induced response following 4 h HS recovery in cells modified and heat-shocked as in **f**. As a positive control (PC), scrambled 15′ IL1β sample from **e** was loaded. **h** Effect of HSF1 knock-down on TNFα-induced NF-κB response in MCF7 cells stably expressing p65-EGFP. Cells treated with scrambled siRNA control (scrambled) and HSF1-specific siRNA (KD HSF1) were either cultured in normal conditions (37 °C, no HS) or subjected to 1 h HS at 43 °C and recovered for 4 h (1 h HS + 4 h). Individual single cell nuclear p65-EGFP trajectories (*n* = 202, 127, 146, 98 respectively, in aribirary fluorescene units) are depicted with blue and violet lines; black lines represented the population averages. Cells monitored for up to 10 h from the beginning of cytokine stimulation. **i** Effect of HSF1 knock-down on IL1β-induced NF-κB response in MCF7 cells stably expressing p65-EGFP. Cells were cultured and stimulated as in **h** (*n* = 153, 140, 113, 42 respectively). For heat maps and number of responding cells see Fig. S2

The cellular response to HS involves the HSF1-dependent transcription of genes encoding HSPs as a part of an internal stress-adaptation mechanism (Fig. 3a). Exposure of MCF7 cells to 1 h HS resulted in hyperphosphorylation of HSF1 (visible as a shift of the HSF1 protein in western blot analysis which coincided with Ser326 phosphorylation, a marker of temperature-induced HSF1 activation; Fig. 3b) [55]. While transient, the HSF1 activation resulted in a robust upregulation of HSPA1, a major factor involved in the internal protein repair [56]. The *HSPA1* mRNA levels showed steady increases for up to several hours after 1 h HS exposure (Fig. 3c), while an increased HSPA1 protein level was observed from the second/third hour of recovery (Fig. 3b).

The observed kinetics of HSPA1 protein accumulation coincided with the timing of the TNFα-induced NF-κB signalling recovery after HS (Fig. 1). We, therefore, sought to directly establish if HSF1 feedback controls these responses. For this purpose, we used siRNA to selectively knock-down the expression of HSF1 in MCF7 cells. The immunoblotting experiments performed both in cells treated with HSF1-specific or scrambled siRNA, confirmed a knock-down of HSF1 expression by siRNA and also revealed the NF-κB activation by TNFα and IL1β in either model (Fig. 3d, e). Subsequently, we exposed cells to 1 h HS and recovered for 4 h before cytokine treatment, a condition that showed differential responses after TNFα and IL1β treatment. Almost complete HSF1 knock-down by siRNA resulted in the inhibition of HSPA1 up-regulation after HS. In cells treated with scrambled siRNA, we observed the recovery of the NF-κB signalling (visible as Ser536 p65 phosphorylation) (Fig. 3f, g) which was essentially the same as in wild type cells (Figs. 1b, 2c). Interestingly, HSF1 knock-down had no influence on the recovery of the NF-κB signalling in response to IL1β stimulation, while the responses to TNFα stimulation did not recover. Further microscopy analyses confirmed that under normal conditions (i.e., 37 °C) cells treated with HSF1-specific or scrambled siRNA showed robust p65-EGFP translocation to the nucleus following both TNFα and IL1β stimulation (Fig. 3h, i, top rows). After HS, markedly reduced p65-EGFP nuclear translocation was observed in cells treated with siRNA specific for HSF1 and stimulated with TNFα but not with IL1β (Fig. 3h, i, bottom rows; see Fig. S2 for heat maps and a fraction of responding cells), which was consistent with differential Ser536 p65 phosphorylation. Overall, this data demonstrates that HSF1-dependent feedback regulates TNFα, but not IL1β-mediated NF-κB responses following HS.

### HS-modulated NF-κB responses are conferred via IKK signalosome

The attenuation of IKK activity via temperature-dependent denaturation and loss of solubility is thought to be critical for the NF-κB responses post HS [31, 32]. We previously showed that exposure of human osteosarcoma cells to 1 h 43 °C HS resulted in depletion of soluble IKKα and IKKβ levels, effectively limiting the amount of IKK (and thus NF-κB) that can be activated by the cytokine stimulation [28]. The cellular adaptation to HS requires a restoration of IKK signalling. However, the kinetics of recovery and the relationship with HSR remain unexplored. Based on our findings that HSF1 is involved in the stimulus-specific NF-κB recovery post-HS, we developed a dynamical mathematical model of the HSR and NF-κB cross-talk to investigate these mechanisms more quantitatively.

**Fig. 4:**
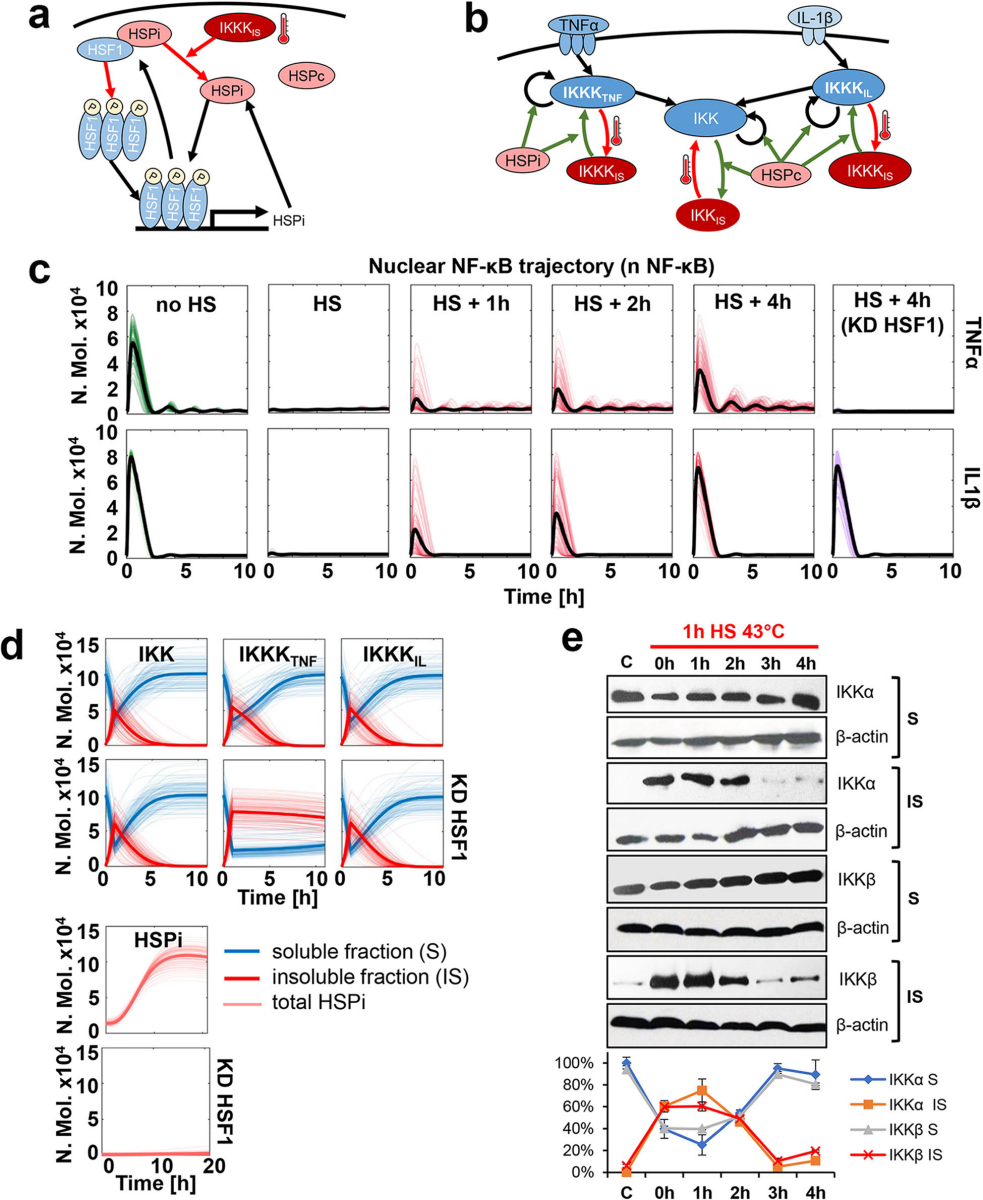
Mathematical model recapitulates HSR and NF-κB interaction via IKK signalosome. **a** Schematic representation of HSF1 signalling system. Red arrows indicate HS dependent regulation. **b** Schematic diagram of the proposed NF-κB and HS pathway crosstalk. Red arrows represent proposed temperature-dependent protein denaturation, green arrows represent events that involve interactions with HSP proteins. **c** Model simulations of the NF-κB-HSR crosstalk: wild type and HSF1 knock-down cells (KD HSF1) treated with TNFα (top) and IL1β (bottom) after different recovery times from HS (as indicated). Shown are the iterations of time-courses of nuclear NF-κB levels (100 representative iterations; coloured lines) and average nuclear NF-κB levels (in black), calculated from 1000 single cell model simulations (in number of molecules). Cells simulated for up to 10 h from the beginning of cytokine stimulation. **d** Kinetics of the IKK signalosome. Simulations were performed for 1 h HS at 43 °C followed by 10 h (IKK and cytokine-specific IKKK) or 20 h (HSPi) recovery time. Shown are time courses of simulated IKK, IKKK and HSPi levels in wild type cells (top) or cells with the HSF1 knock-down (KD HSF1) in number of molecules post HS. **e** Western blot analysis of soluble (S) and insoluble (IS) IKKα and IKKβ proteins level in MCF7 cells, either cultured under normal conditions (C) or subjected to 1 h HS at 43 °C and/or recovered for 1–4 h. β-actin was used as a loading control. The graph below shows the percentage of soluble and insoluble (S + IS = 100% in each experimental point) IKKs calculated based on Western blot densitometry

We considered a simplified structure of the HSR pathway (Fig. 4a), which contains HSF1 and two HSP species: inducible (HSPi), transcription of which is strictly HSF1-dependent, and constitutive (HSPc) [57]. Following previous work, we made a simplifying assumption that in resting cells HSF1 monomers are held in an inactive state via an association with HSPi, while HSPc acts as a generic chaperone for other proteins [7, 45, 46]. Temperature stimulation results in redistribution of HSPi (and HSPc) from the HSF1 complex, then HSF1 forms trimers and activates transcription of HSPi, creating a regulatory feedback loop that restores proteome homeostasis and eventually inhibits HSF1 activity [2]. In addition, we utilised our existing model of the IKK-NF-κB signalosome [15] involving TNFα and IL1β transduction pathways, each comprising a cognate receptor and upstream kinase (IKKK, Inhibitory κB kinase kinase) that in parallel regulate IKK activity (Fig. 4b). As in previous models [15, 54], cytokine-specific IKKKs denote generic IKK kinases, and simplistically represent complex and not fully elucidated signal transduction networks [16, 17, 58]. Subsequently, we considered different crosstalk mechanisms that could recapitulate the kinetics of cytokine-specific NF-κB responses after HS (for details of the mathematical modelling, see the Materials and methods section). Assuming that HS exposure results in ubiquitous damage of the proteins involved in the IKK signalosome, our data (Fig. 3) demonstrated that (1) the recovery of IL1β signalling was independent of the HSF1-mediated response, potentially mediated via the action of constitutive HSPs; (2) the recovery of TNFα signalling depended on the inducible HSF1-HSPi response; which (3) was mediated via the signal-specific pathways upstream of IKK kinase (IKKK_TNF_). These mechanisms, when implemented in the combined cross-talk model, were able to very closely recapitulate the experimentally observed NF-κB responses to TNFα and IL1β stimulation, in wild type and HSF1 knock-down cells (Fig. 4c, see also Tables S2, S3 and S4 for model equations and fitted parameter values of the IKK-HSP interaction).

In the mathematical model, NF-κB behaviour is dictated by the changing levels of IKK activity [14]. Simulations suggest that the peak of denatured (insoluble) IKK occurs immediately after HS (at 1 h), then it fully recovers to intact (soluble) forms following 4 h post-HS (Fig. 4d). The IL1β-specific IKK kinase (IKKK_IL_) recovers with similar kinetics. In contrast, TNFα-specific IKK kinase (IKKK_TNF_) recovers more slowly, which dictates the overall NF-κB signalling recovery post-HS. In order to verify these predictions, we measured by immunoblotting the levels of soluble (intact) and insoluble (denatured) levels of IKKα and IKKβ subunits in MCF7 cells exposed to 1 h HS and recovered for up to 4 h (Fig. 4e). In lysates from cells cultured under normal conditions, IKKα and IKKβ were detected only in the soluble form. 1 h HS exposure resulted in the transition to insoluble forms, indicating their temperature-induced denaturation. The amount of insoluble IKKα and IKKβ fractions remained high up to 2 h after HS exposure, but effectively returned to pre-HS state at 3 h after exposure in agreement with model simulations. In addition, we investigated whether recovery after HS might depend on the cognate receptor availability and internalisation [59]. Cells were stimulated with fluorescently labelled TNFα or IL1β and observed under the microscope. In agreement with our modelling assumptions, no difference in the binding and internalisation of either cytokine was detected in cells exposed to HS in comparison to cells cultured under normal temperature (Fig. S3). Overall, these analyses indicated that hypothetical stimulus-specific signallig via IKK signalosome (e.g., the activity of cytokine-specific IKK kinases) differently modifies the NF-κB response after HS, which in part depended on inducible HSF1 responses.

### NF-κB sensitivity to temperature of HS is stimulus-specific

Mammalian cells experience a wide range of temperatures from physiological core body temperature and fever (< 40 °C) to heat shock used in clinical hyperthermia treatment (up to 45 °C) [50]. Having established the critical link between HSR and the NF-κB systems, we wanted to understand their sensitivities to a range of relevant temperatures. First, we assayed IKK solubility (Fig. S4A). The 1 h exposure to different temperatures in a 38–43 °C range resulted in increased levels of insoluble IKKα and IKKβ (in comparison to cells cultured at 37 °C) and decresed levels of soluble IKKs in cell lysates (in particular at 43 °C). In addition, we used live-cell images of MCF7 cells expressing HSF1-dsRed to measure the HSR activation by examining the redistribution of the fusion protein into nuclear stress granules [60] (Fig. S4B). We found a significant increase in the number of HSF1-dsRed granules at 41 °C, compared to lower temperatures and the 37 °C control, which further increased at 42 °C (Fig. S4C), that matched the temperature-dependent shift of the total HSF1 protein in the immunoblotting assay (Fig. S4D). Overall these data indicated possible temperature-dependent modulation of the NF-κB response over the 38–43 °C range, with HSF1 response activated above 40 °C. Our mathematical model was subsequently extended to incorporate the observed temperature-dependent IKK and HSF1 behaviour (Fig. S5). For simplicity, we assumed denaturation of IKK followed a nonlinear temperature-depended function and receptor-specific IKKKs shared the same characteristics (see Tables S3 and S4 for model description). Under these assumptions, 1 h exposure to increasing temperatures resulted in the gradual decrease of the soluble IKK and cytokine-specific IKKK levels (Fig. S5A). In turn, the graded protein denaturation resulted in a step-like HSF1 activation as a consequence of HSF1 redistribution form the HSP-HSF1 complex (Fig. S5B). As such, the model suggested that the level of IKK denaturation was closely linked to the level of HSF1 activation in the system (Fig. S5C).

**Fig. 5:**
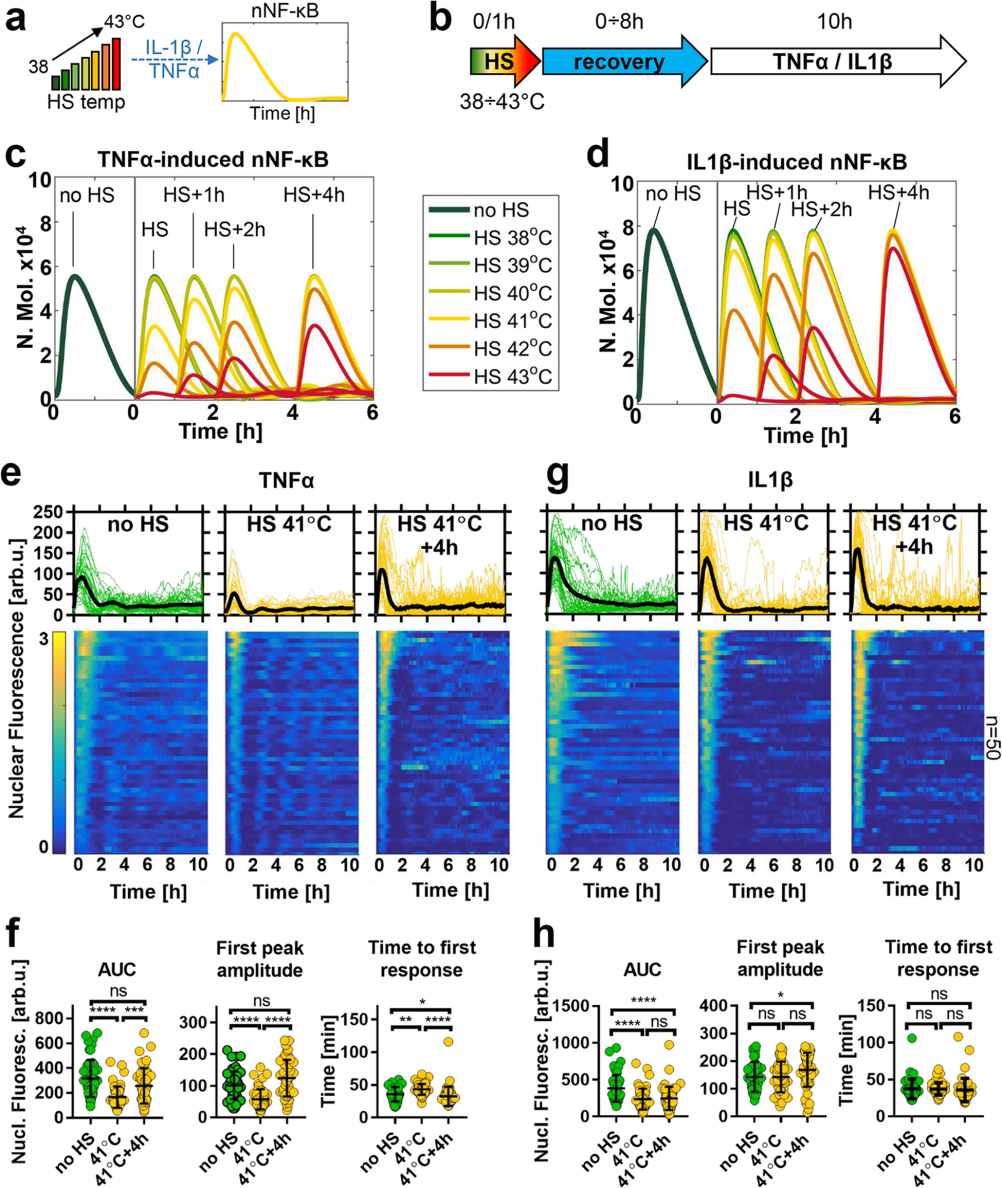
NF-κB responses exhibit cytokine-specific temperature sensitivity. **a** Schematic representation of the differential temperature treatment. **b** Schematic representation of the HS treatment protocol: cells exposed to 1 h 38–43 °C HS range and subjected to cytokine stimulation following different recovery time. **c** Model simulations of the TNFα-induced NF-κB responses following 1 h temperature exposure and different recovery times. Shown are average nuclear NF-κB trajectories (based on 1000 simulated cells, in number of molecules) for the 38–43 °C temperature range and normal 37 °C conditions (in diffrent coloured lines); the right 0–6 h axis represents time from the end of HS, on the left: no HS control. **d** Model simulations of the IL1β-induced responses as described in **c**. **e** Nuclear NF-κB trajectories in MCF7 cells stably expressing p65-EGFP in response to TNFα stimulation. Cells were stimulated with TNFα in normal temperature 37 °C (left, data from Fig. 1), immediately after 1 h 41 °C exposure (middle), or after 4 h recovery (right). Top: individual single cell nuclear NF-κB trajectories (*n* = 50 per condition, in aribirary fluorescene units) depicted with coloured lines (green 37 °C, yellow 41 °C); black lines represented the population averages. Bottom: heat maps of trajectories normalized across all conditions in **e** and **f** (represented on a 0–3 scale). Cells monitored for up to 10 h from the beginning of cytokine stimulation. **f** Characteristics of TNFα-induced responses from **e**. From the left: distribution of area under the curve (AUC), first peak amplitude, and time to first response. Individual cell data are depicted with circles (with mean ± SD per condition). Kruskal-Wallis one-way ANOVA with Dunn’s multiple comparisons test was used to assess differences between groups (*p < 0.05, **p < 0.01, ***p < 0.001, ****p < 0.0001*,* ns – not significant). **g** Nuclear NF-κB trajectories in MCF7 cells stably expressing p65-EGFP following treatment with IL1β, represented as in **e**. p65-EGFP trajectories of cells cultured in normal conditions taken from Fig. 2. **h** Characteristics of IL1β-induced responses from **g**, data are represented as in **f**

Subsequently, using the developed model we performed comprehensive simulations to understand NF-κB attenuation and recovery following different temperature exposures (Fig. 5a and b). We found that in the case of the TNFα treatment, the model exhibited NF-κB inhibition following exposure to 41 °C or higher temperatures (compared to cells cultured under normal condition) (Fig. S6A). Immediately after 1 h exposure to 41 °C, the first peak nuclear NF-κB amplitude was 56% of that in control cells, while exposure to 42 °C showed further inhibition to 31% (Fig. 5c). In contrast, IL1β-induced NF-κB signalling was predicted to be less sensitive to temperature changes; no inhibition was observed at 41 °C, and only a partial inhibition was observed after 1 h exposure to 42 °C HS (Fig. 5d and Fig. S7A). In order to validate these predictions, we performed live-cell imaging studies at the critical 41 °C temperature. In agreement with modelling, we found an inhibition of TNFα-induced responses in cells stimulated immediately after 1 h 41 °C exposure (i.e. significant reduction of AUC and peak amplitude as well as increased time to first response, compared to cells cultured under normal condition). As predicted, responses returned to pre-stimulation steady-state in cells stimulated after 4 h recovery (Fig. 5e and f). Also, we found no inhibition IL1β-mediated NF-κB responses by 41 °C (in particular immediately after the exposure), validating the prediction of stimulus-specific temperature sensitivity (Fig. 5g and h). In the mathematical model, the increased sensitivity of TNFα-induced responses to HS was due to a lower NF-κB amplitude and thus IKK activity (in comparison to IL1β-induced response), which in turn was more affected by the level of protein denaturation at a given temperature (Figs. S5D, S6B and S7B). Systematic sensitivity analyses confirmed that the kinetic parameters associated with the IKK module (but not IκBα feedback) control differential NF-κB responses to temperature changes (Fig. S8). This analysis also revealed a role in temperature regulation for the A20 protein feedback acting via IKK on temperature sensitivity in line with previous reports [27].

### Timing of HSP induction renders adaptation to repeated temperature stress

**Fig. 6:**
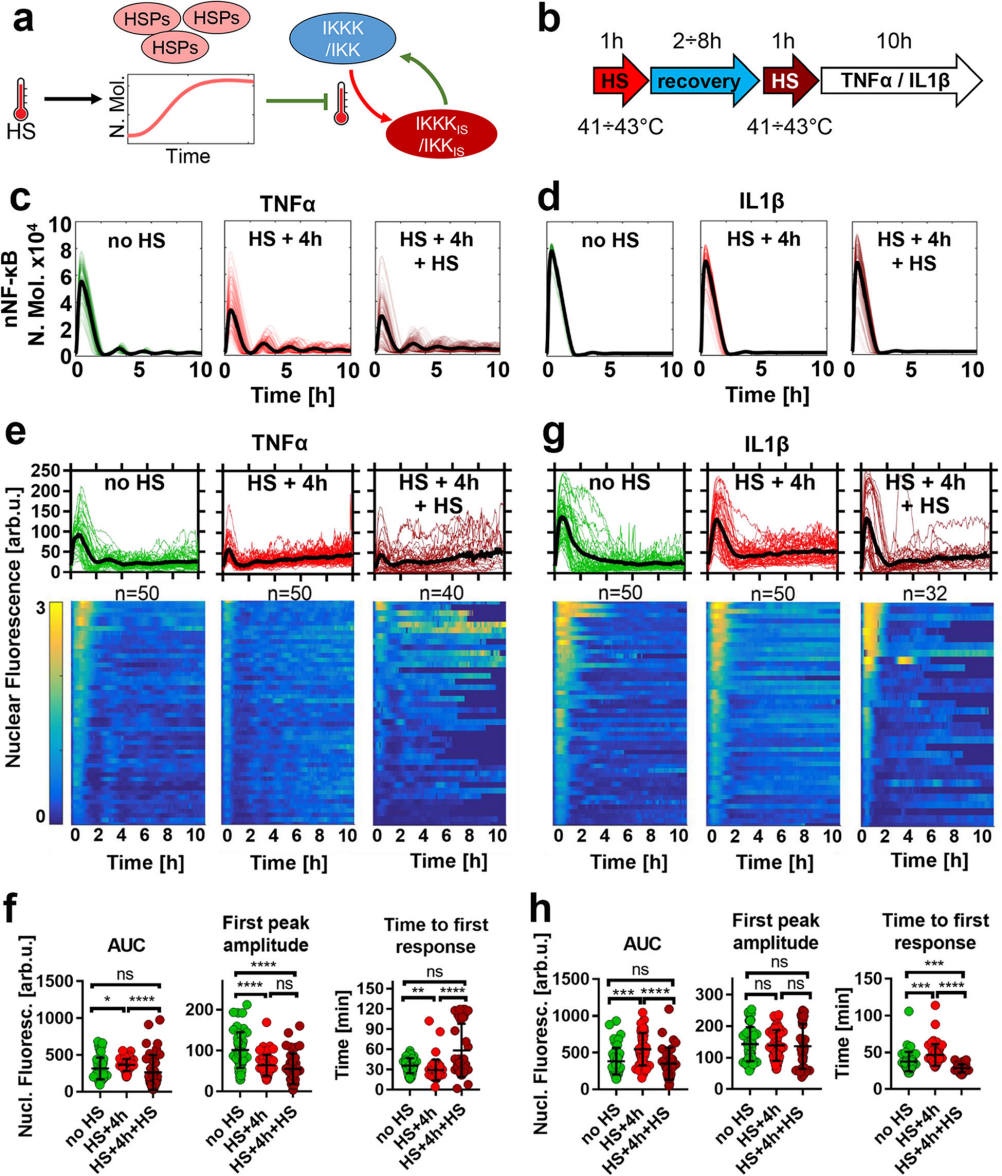
NF-κB adaptation to repeated temperature exposure. **a** Schematic representation of the thermotolerance: accumulation of inducible HSPs prevents IKK denaturation to repeated HS exposure. **b** Schematic representation of the repeated HS treatment protocol: cells exposed to two 1 h HS at indicated interval and subjected to cytokine stimulation. **c** Model simulation of TNFα-induced NF-κB responses following different HS protocols. Shown are sample 100 trajectories of single cell (and average responses based on 1000 simulated cells, in number of molecules, in black) treated in normal temperature 37 °C (left, in green), 4 h after a single 1 h 43 °C HS exposure (middle, in red) and immediately after second 1 h 43 °C HS exposure (right, in burgundy). **d** Model simulation of IL1β-induced NF-κB responses following different HS protocols, as in **c**. **e** Nuclear NF-κB trajectories in MCF7 cells stably expressing p65-EGFP in response to TNFα stimulation. Cells are either treated in normal conditions (left, data from Fig. 1), 4 h after a single 1 h 43 °C exposure (middle, data from Fig. 1) or immediately after second 1 h 43 °C HS exposure (right). Top: individual single cell nuclear NF-κB trajectories (n = 50, 50 and 40 per condition, respectively, in aribirary fluorescene units) depicted with coloured lines; black lines represented the population averages. Bottom: heat maps of trajectories normalized across all conditions in **e** and **f** (represented on a 0–3 scale). Cells monitored for up to 10 h from the beginning of cytokine stimulation. **f** Characteristics of TNFα-induced responses from **e**. From the left: distribution of area under the curve (AUC), first peak amplitude, and time to first response. Individual cell data are depicted with circles (with mean ± SD per condition). Kruskal-Wallis one-way ANOVA with Dunn’s multiple comparisons test was used to assess differences between groups (*p < 0.05, **p < 0.01, ***p < 0.001, ****p < 0.0001*,* ns – not significant). **g** Nuclear NF-κB trajectories in MCF7 cells stably expressing p65-EGFP following treatment with IL1β, represented as in **e**. p65-EGFP trajectories of cells cultured in normal conditions or exposed to IL1β after 4 h recovery from 1 h 43 °C are taken from Fig. 2. **h** Characteristics of IL1β-induced responses from **g**, data are represented as in **f**

Overall, our analyses of cells exposed to elevated temperatures demonstrate that the HSF1-NF-κB crosstalk enables stimulus-specific responses to pro-inflamatory cytokines with differential temperature sensitivity. However, cells are also known to be able to adapt to repeated temperature challenges [10]. This so-called thermotolerance effect is thought to depend on HSP accumulation [31, 32], which once induced may prevent IKK (and general proteome) damage to subsequent temperature exposures (Fig. 6a). Our data demonstrated that inducible HSPA1 accumulated in the system after 2-3 h following exposure to 43 °C (Fig. 3b), while simulations suggested accumulation over the 41–43 °C range (Fig. S5E). We, therefore, used our crosstalk model to simulate NF-κB system responses following exposures to repeated temperature treatments at different time intervals ranging from 2 to 8 h (Fig. 6b). We found that in the case of TNFα stimulation, exposure to elevated temperatures at 2 h time interval resulted in NF-κB response inhibition in the 41–43 °C range (Fig. S9A), when compared to a single HS exposure (Fig. S6). Thermotolerance, i.e. lack of NF-κB inhibition in cells exposed to repeated temperature treatment, was predicted to occur as early as 4 h after the initial exposure (Fig. 6c). The IL1β-induced responses were predicted to be affected only at 2 h exposure interval and only at 42 and 43 °C temperatures (Fig. S9B), while responses at 4 h were fully adapted (Fig. 6d). As before we performed live-cell imaging studies to validate these predictions. We found that at the critical 43 °C temperature, cells treated with TNFα were adapted to the second temperature treatment applied 4 h after the first HS. In this case, the amplitude of the first peak of nuclear p65-EGFP could not be distinguished from that in cells treated 4 h after a single HS exposure (Fig. 6e and f). Of note, some cells exhibited delayed response times (comparing to a single HS treatment or in fact cells treated under normal conditions) highlighting increased heterogeneity of the responses. Also, as predicted by our mathematical model, IL1β-induced responses following repeated 43 °C exposure were fully adapted, i.e. nuclear p65-EGFP amplitude was similar to that of a single HS treatment as well as of cells treated in normal conditions (Fig. 6g and h). In the mathematical model, the apparent HSP accumulation prevented IKK signalosome damage to the second HS exposure facilitating a normal cytokine-induced response (Fig. S9A and S9B). In agreement, immunoblotting analyses demonstrated a lack of IKKα and IKKβ denaturation following exposure to repeated HS after 3 h recovery (Fig. S9C). Overall, these analyses validate the predictive power of our mathematical model and demonstrate that via the HSR feedback the NF-κB system may rapidly adapt to repeated temperature treatment.

## Discussion

Temperature perturbs key cellular processes and cell function, in particular, involved in proliferation and inflammatory signalling [61]. Thus, the ability to sense temperature changes to restore homeostasis is one of the most fundamental cellular response systems [2]. Here we provide a new quantitative understanding of the dynamical crosstalk mechanisms involved in the regulation of the HSR and NF-κB networks using live single-cell microscopy and mathematical modelling approaches. We demonstrate that the kinetics of the NF-κB system following temperature stress is cytokine-specific and may exhibit rapid adaptation to temperature changes. In agreement with previous findings, the exposure of breast adenocarcinoma cells to 43 °C HS resulted in the attenuation of the immediate NF-κB signalling and gene expression response to TNFα and IL1β stimulation. However, while the IL1β-induced responses return to the normal level within 4 h after HS exposure, the recovery following TNFα-mediated responses is delayed. TNFα and IL1β are two cytokines that confer related but distinct pro-survival and pro-inflammatory functions. Their expression is elevated in the local or inflamed tissue, e.g. in breast cancer [62], and their signalling, in the context of elevated temperature is important for physiological and clinical cellular responses [11]. Previous studies have focused solely on TNFα-mediated NF-κB signalling [25, 28–30, 32], or examined a combined effect of cytokine mixes [31]. Here we show that the stimulus-specificity is rendered via HSR differentially controlling TNFα and IL1β signal transduction pathways. We argue that these data essentially reveal that (1) the recovery of IL1β signalling is independent of the inducible HSF1 response, potentially mediated via the action of constitutive HSPs, (2) the recovery of TNFα signalling depends on the inducible HSF1-HSPi response, which (3) is mediated via the signal-specific pathways upstream of the IKK signalosome. Moreover, we demonstrate that individual cytokines essentially exhibit different temperature sensitivity and adaptation to repeated HS when exposed to a 37–43 °C temperature range. Specifically, IL1β-mediated NF-κB responses are more robust to temperature changes in comparison to those induced by TNFα treatment.

Several lines of evidence highlight the modulation of IKK signalosome and the concurrent HSF1-HSP feedback activation as the key regulators of responses to elevated temperature [1, 24, 25, 28–30, 32, 39]. It is thought that physiological temperatures (< 40 °C) modulate IKK and consequently fine-tune NF-κB responses (oscillations) via the timing of the A20 negative feedback [27]. In contrast, responses to extreme temperatures (> 40 °C) have been shown to involve direct attenuation of IKK activity via temperature-dependent denaturation and loss of solubility [28, 31–33]. Consequently, the temporal adaptation to HS requires a restoration of IKK and upstream transduction pathways [63]. Consistently with this idea, here we observe the temperature-dependent gradual loss of IKKα and IKKβ solubility over a 38–43 °C range, levels of which return to the resting steady state within 6 h post-HS. We fully expect that several additional mechanisms might contribute to the observed behaviour [28]. Importantly, the regulatory IKKγ subunit was also shown to undergo denaturation similar to that of IKKα and IKKβ post-HS [33], therefore further modulating kinase activity (in addition to protein level) [17]. Additional mechanisms might include inhibition of NF-κB transport (via cytoskeletal alternations) [38] or level [41], transcriptional co-regulation of NF-κB-dependent genes by HSF1 [30] and in particular, diverse action of specific HSP molecules [64]. In turn, it would be interesting to investigate the extent of the reciprocal cross-talk mechanisms in the system (e.g. effect of NF-κB signalling on HS activation [30]), which could be exhibited by differential IKK recovery under different cytokine stimulation protocols.

When it comes to signal specificity upstream of IKK and delayed recovery of TNFα-induced responses, each transduction pathway acts in parallel. TNFα signalling involves TRAF2/RIP, while IL1β involves TRAF6/IRAK-mediated pathways via enzymatic interactions with A20 [17, 58]. The latter is shared with the toll-like receptor signalling [16], suggesting similar responses during infection. The mechanisms acting in their differential HSR control remains to be elucidated. One interaction that might be important in HSF1-dependent modulation of TNFα signalling is the apparent temporal sequestration of TRAF2 adaptors into stress granules following HS [65], and thus the requirement for their recovery. In this work, using live single-cell microscopy we have the ability to monitor the heterogeneity of NF-κB signalling responses [66]. While responses appear to be more homogenous than those reported in the previous work using human osteosarcoma cells [28], we observe that TNFα-induced oscillatory patterns become more robust following recovery from HS in comparison to cells treated under normal conditions. As such, this is consistent with the idea that inducible HSPs (given their long half-lives) [39] might be able to influence NF-κB responses and potentially target gene expression over prolonged periods.

The ultimate goal of mathematical modelling is to interpret data and make biological predictions [67]. Here we developed and validated a dynamical mathematical model of NF-κB and HSR crosstalk, which combines previously published network structures [7, 15, 45, 46]. We proposed a crosstalk mechanism to recapitulate our original data on NF-κB responses in wild type and HSF1 knock-down cells as well as measurements of the IKK denaturation and activation of the HSF1-HSP pathway for a 37–43 °C temperature range. Here, we make simplifying assumptions, which essentially allow us to directly relate the level of IKK damage to the level of HSF1 activation for a given temperature suggesting that the former is indicative of the overall proteome damage. Using our developed mathematical model, we systematically screened the NF-κB signalling responses to single and repeated HS exposures for a range of temperatures and recovery times. We predicted critical conditions where the TNFα and IL1β-mediated responses exhibit differential sensitivity to temperature or HSP-mediated thermotolerance. Subsequently, we performed additional imaging experiments to validate these predictions. These specifically demonstrate that the TNFα-induced NF-κB signalling responses are attenuated at 41 °C, while the corresponding IL1β-induced signalling remains intact. In combination with sensitivity analyses this reinforces the idea that the IKK signalosome is a bona fide temperature sensor [68], which effectively enables NF-κB signalling responses with cytokine specificity and differential temperature sensitivity. It will be important to understand the biophysical basis for the apparent temperature sensitivity of IKK subunits and molecules involved in signal transduction. Of note, we previously showed that the NF-κB appears to be more stable than IKK molecules and does not undergo denaturation with 1 h 43 °C HS [28]. Whether in general the signal transduction molecules are more temperature-sensitive than other network components would be important to understand [69].

The kinetic HSR and the NF-κB crosstalk is relevant to hyperthermia, an emerging therapeutic strategy to sensitise cancer cells to cytotoxic treatment (chemotherapy or radiotherapy) with an artificial increase of tissue temperature [26]. The efficacy of hyperthermia treatment is thought to critically depend on the timing between the HS exposure and treatment, to maximise the effect of proteome damage and minimise the effect of prosurvival induction of thermotolerance [46]. Current treatment protocols utilise a wide time window, where HS in the range of 38–45 °C is applied for up to 24 h before and after treatment [50]. Our analyses demonstrate that the time windows rendering cells sensitive to treatment may be short even at extreme temperatures, i.e. up to 4 h at 43 °C, and even absent at temperatures below 41 °C. Moreover, one should expect that in different cell/tissue types the NF-κB system (or the combined action of other systems, including MAP kinase [70] or p53 tumour suppressor [71]) might exhibit different sensitivities and recovery kinetics following temperature exposure. For example, we previously showed that TNFα stimulation in human osteosarcoma cells resulted in “all-or-nothing” NF-κB responses following HS [28], while tissue-level architecture might impose additional spatial constraints [53]. We suggest that further efforts should combine dynamical modelling with cell fate, in order to better understand the relationship between temperature and NF-κB as well as cell proliferation and apoptosis in the more relevant cancer or inflammatory context. Nevertheless, it is clear that clinically effective hyperthermia protocols require optimization based on a quantitative understanding of the underlying processes.

## Conclusions

Here we provide a new quantitative understanding of the dynamical crosstalk mechanisms involved in the regulation of the HSR and NF-κB networks using live single-cell microscopy and mathematical modelling approaches. We demonstrate that the kinetics of the cytokine-induced NF-κB system following temperature stress is stimulus-specific and exhibit differential adaptation to temperature changes. Specifically, our results indicate that TNFα-mediated signalling is inhibited by elevated temperatures more effectively than IL1β-mediated signalling. Moreover, the post-HS recovery is controlled via HSF1-regulated pathways only in the case of TNFα signalling. The new knowledge of the crosstalk between HSR and NF-κB could help to understand physiological processes related to fever and optimizing therapeutic protocols involving hyperthermia.

### Acknowledments

We thank Michael White and other members of Systems Microscopy Centre in Manchester for discussions.

## Supplementary information

**Additional file1 : Figure S1.** Analysis of NF-κB signalling responses in MCF7 cells. (**A**) Analysis of NF-κB p65-Ser536 phosphorylation in transformed cells. The level of p65-Ser536 phosphorylation was analyzed by Western blot in the whole MCF7 p65-EGFP cells lysates. Cells cultured in 37 °C were treated with TNFα for indicated times. β-actin was used as a loading control. (**B**) Nuclear NF-κB trajectories in MCF7 cells stably expressing p65-EGFP after 1 h 43 °C HS treatment. Individual single cell trajectories (*n* = 50 per condition) are depicted with colour lines; population average is depicted with a black line. (**C**) Quantitative RT-PCR analysis of *TNFAIP3*, *NFKBIA*, *CCL2,* and *TNF* mRNA abundance 90 min after TNFα or IL1b stimulation of cells cultured at 37 °C (no HS) or following HS treatment and indicated recovery time. Shownare mean changes in relation to unstimulated cells ± SDs based on three replicate experiments. **Figure S2.** Analysis of NF-κB responses in HSF1 knock-down cells. (**A**) Heat maps of nuclear NF-κB trajectories in response to TNFα in MCF7 cells stably expressing p65-EGFP. Cells were treated with scrambled siRNA control (scrambled) or HSF1-specific siRNA (KD HSF1) and stimulated with the cytokine under normal conditions (37 °C, no HS) or after 1 h HS at 43 °C and 4 h recovery (1 h HS + 4 h). Heat maps of trajectories were normalized across all conditions (represented on a 0–3 scale). Individual single cell trajectories are shown. (**B**) Heat maps of nuclear NF-κB trajectories in response to IL1β in MCF7 cells stably expressing p65-EGFP. Cells were treated and data are presented as in A. (**C**) Percentage of cells responding (yellow) and non-responding (blue) to stimulation with TNFα or IL1β (from data shown in A and B). Statistical difference was assessed with Chi-square test (****p < 0.0001, ns – not significant). **Figure S3.** Analysis of cytokine uptake after HS. (**A**) Confocal microscopy images of representative MCF7 cells stimulated with fluorescently labelled TNFα. Cells were cultured under normal conditions (at 37 °C, no HS) or exposed to 1 h HS at 43 °C prior to TNFα stimulation. FITC-conjugated TNFα was applied at 0 min and measured 10 min after stimulation. Top – bright field, middle – FITC, bottom – merged images. Scale bar, 10 μM. On the right: quantified individual cell fluorescent levels as well as mean ± SD per condition, based on three experimental replicates. (**B**) Confocal microscopy images of representative MCF7 cells stimulated with fluorescently labelled IL1β and quantified fluorescence levels (as in A). **Figure S4.** Temperature sensitivity of the NF-κB and HSR signalling. (**A**) Western blot analysis of soluble (S) and insoluble (IS) IKKα and IKKβ proteins level in MCF7 cells. Cells were either cultured under normal conditions, 37 °C, or subjected to 1 h temperature shift (38–43 °C range, as indicated on the graph). β-actin was used as a loading control. (**B**) Temperaturesensitivity of HSF1 stress granule formation. Confocal microscopy images of representative MCF7 cells stably expressing HSF1-dsRed fusion protein. (Top) Cells cultured under normal conditions (at 37 °C, no HS) or exposed to 1 h HS at 43 °C and imaged thereafter. Recovery time after HS is displayed in minutes. Scale bar 5 μm. (Bottom) Cells assayed under normal conditions (at 37 °C, no HS) or assayed following 1 h HS at 38–43 °C temperature range. Scale bar 10 μm. (**C**) Distribution of stress granules in MCF7 cells stably expressing HSF1-dsRed. Individual cell data as in B are depicted with circles (with mean ± SD per condition, of > 117 cells per condition). Kruskal-Wallis one-way ANOVA with Dunn’s multiple comparisons test was used to assess differences between groups (****p < 0.0001*,* ns – not significant). (**D**) Western blot analysis of the total HSF1 protein level in MCF7 cells. Cells were either cultured in normal conditions, C, or subjected to 1 h temperature stress in the 38–43 °C range. β-actin was used as a loading control. Shift of the HSF1 band indicates activation. **Figure S5.** Temperature sensitivity of the IKK and HSF1 in the mathematical model (**A**) Comparison of simulated soluble/insoluble IKK and IKKK kinase fractions after 1 h HS assuming a 38–43 °C temperature range (as indicated on the graph). 37 °C represents cells cultured under normal conditions. Shown are average protein levels and standard deviations calculated based on 1000 single cell model simulations (in number of molecules). (**B**) Simulated level of active HSF1 under conditions as in A. (**C**) A comparison of the peak active IKK kinase level and active HSF1 as a function of temperature. Shown are average protein levels, calculated from 1000 single cell model simulations (in number of molecules), following TNFα and IL1β treatment immediately after 1 h HS exposure. (**D**) Differential cytokine sensitivity to temperature: temperature-dependent depletion of soluble IKK following HS (left) affects TNFα-induced IKK activity (transition from resting inactive, IKKn to active form, IKKa) more than that of IL1β, due to its lower activation amplitude (right). Shown are averages of 1000 simulated cells (in number of molecules) treated with cytokine immediately after 1 h HS exposure to the indicated temperature range. (**E**) Kinetic of HSPi protein accumulation depends on the HS temperature. Shown are average HSPi levels, calculated from 1000 single cell model simulations after 1 h HS at different temperatures. **Figure S6.** Model simulations of TNFα-induced responses following range of HS temperatures and different recovery times. (**A**) Cells are exposed to 1 h HS from a temperature range and recovered for up to 8 h before cytokine stimulation. Shown are sample 100 time-courses of nuclear NF-κB levels (coloured lines) and average nuclear NF-κB levels (in black), calculated from 1000 single cell simulations (in number of molecules). (**B**) Comparison of IKK and IKKK kinase levels in simulated data from A. **Figure S7.** Model simulations of IL1β-induced responses following range of HS temperatures and different recovery times. (**A**) Cells are exposed to 1 h HS from a temperature range and recovered for up to 8 h before cytokine stimulation. Shown are sample 100 time-courses of nuclear NF-κB levels (coloured lines) and average trajectory (in black), calculated from 1000 single cell simulations (in number of molecules). (**B**) Comparison of IKK and IKKK kinase levels in simulated data from A. **Figure S8.** Temperature sensitivity analysis of the NF-κB signalling network. Shown are heat maps describing the influence of model parameters (listed in the table below) involved in (**A**) IKKK, (**B**) IKK, (**C**) A20 and (**D**) IκBα regulation for a range of HS temperatures. All results show sensitivity index calculated for the average nuclear NF-κB levels in the first peak based on 1000 single cell simulations, normalised to 0–1. Vertical changes indicate increased sensitivity to temperature, nominal parameter values for TNFα and IL1β transduction pathways are indicated with broken lines. **Figure S9.** Responses to repeated HS treatment. (**A**) Model simulations of cells exposed to repeated 1 h HS from a temperature range at a different time interval (from 2 to 8 h) and treated with TNFα (immediately after the second HS exposure). Shown are sample 100 time-courses of nuclear NF-κB levels (coloured lines) and average trajectory (in black), calculated from 1000 single cell simulations across conditions (in number of molecules). Bottom: comparison of the corresponding IKKKTNF kinase levels following different treatment protocols. (**B**) Simulation of responses to IL1β, following the protocol described in A. (**C**) Western blot analysis of soluble (S) and insoluble (IS) IKKα and IKKβ proteins level in MCF7 cells. Cells were either cultured under normal conditions, 37 °C, subjected to 1 h 43 °C HS or subjected to repeated HS after 3 or 4 h (as indicated on the graph). β-actin was used as a loading control. **Table S1.** RT-qPCR primer sequences used in the study. **Table S2.** Mathematical model variables. **Table S3.** Mathematical model equations. **Table S4.** Model parameters.

## Data Availability

The datasets and mathematical model codes analysed during the current study available from the corresponding author on reasonable request.

## Abbreviations

HS: Heat Shock
HSF1: Heat Shock Factor 1
HSP: Heat Shock Proteins
HSPi: induced HSP
HSPc: constitutive HSP
HSR: Heat Shock Response
IKK: Inhibitory κB kinase
IKKK: inhibitor of nuclear factor κB kinase kinase
IKKK_IL_: IL1β-specific IKK kinase kinase
IKKK_TNF_: TNFα-specific IKK kinase kinase
NF-κB: Nuclear Factor κB
